# The politics of decarbonization and the catalytic impact of subnational climate experiments

**DOI:** 10.1007/s11077-018-9314-8

**Published:** 2018-03-07

**Authors:** Steven Bernstein, Matthew Hoffmann

**Affiliations:** Department of Political Science, University of Toronto, Toronto, Canada

**Keywords:** Climate governance experiments, Climate policy, Global climate regime, Decarbonization, Subnational climate politics

## Abstract

The Paris Agreement of 2015 marks a formal shift in global climate change governance from an international legal regime that distributes state commitments to solve a collective action problem to a catalytic mechanism to promote and facilitate transformative pathways to decarbonization. It does so through a system of nationally determined contributions, monitoring and ratcheting up of commitments, and recognition that the practice of climate governance already involved an array of actors and institutions at multiple scales. In this article, we develop a framework that focuses on the politics of decarbonization to explore policy pathways and mechanisms that can disrupt carbon lock-in through these diverse, decentralized responses. It identifies political mechanisms—normalization, capacity building, and coalition building—that contribute to the scaling and entrenchment of discrete decarbonization initiatives within or across jurisdictions, markets, and practices. The role for subnational (municipal, state/provincial) climate governance experiments in this new context is especially profound. Drawing on such cases, we illustrate the framework, demonstrate its utility, and show how its political analysis can provide insight into the relationship between climate governance experiments and the formal global response as well as the broader challenge of decarbonization.

There is no global climate regime, at least in the terms and form commonly understood over the past 25  years—a top-down, centralized, legal global response based on a treaty developed through multilateral negotiations. The Copenhagen Accord of 2009 hinted at a seismic shift by introducing the world to nationally based commitments substituting for collectively agreed greenhouse gas (GHG) emissions reduction targets. The Paris Agreement of 2015 cemented the transformation. Multilateral climate governance is now a combination of nationally determined contributions (NDCs) and international monitoring, verification, with (one hopes) pressure for ratcheting up those national contributions over time to meet an aspirational target of holding global warming well below two degrees centigrade. The Paris Agreement for the first time explicitly recognizes that *all* states must come up with plans to cumulatively contribute to achieving this goal, which essentially entails functional decarbonization in the second half of this century. Moreover, it acknowledges that much of the actual work of decarbonization required will be undertaken or catalyzed by a wide array of other actors, including subnational (e.g., municipal, state/provincial) and non-state (Hale 2016).

As important as this shift in legal form and institutional structure is—variously described in terms like “bottom up” and “regime complex” (Sabel and Victor 2017; Falkner et al. 2010; Keohane and Victor 2011; Falkner 2016)—the Paris Agreement embodies at least the beginnings of an even more profound conceptual shift in how we understand the problem of climate change: from a legal regime conceived as a negotiated distribution of commitments to solve a collective action problem (reduce emissions) to preserve a global commons (a stable climate) to a catalytic mechanism to promote and facilitate transformative pathways to decarbonization. There is no longer a single focus or locus of global climate action. Decarbonization requires a range of actions from diverse actors. Rayner (2010) hinted at this shift when he called for a move away from viewing climate change governance as an “optimization” problem obsessed with “free riders” and “leakage”.

While this conceptual shift remains incomplete, it is our starting point for thinking about the role and contribution of subnational actors and actions. As opposed to others who view substate and non-state actions as filling the “gap” between NDCs and the emissions levels necessary to reach the Paris Agreement’s aspirational goal,^1^ we view them as experiments in the new context the Paris Agreement acknowledges—a decentralized, fragmented global response to climate change designed to decarbonize societies (Jordan et al. 2015; Zelli and van Asselt 2013; Bulkeley et al. 2014).

Seen in this light, the key question for analysis is not how they fill emission “gaps,” but how subnational (among other types of) climate governance experiments^2^ disrupt carbon lock-in (Unruh 2000; Seto et al. 2016) and promote decarbonization pathways. Emissions are the symptom of the underlying carbon lock-in problem. Subnational experiments can play a key role in achieving that goal because of the nature of carbon lock-in. Globally, economic, energy, and transportation systems are locked into carbon because they are also locked into carbon locally, regionally, and nationally. Subnational experiments may have the potential to disrupt carbon lock-in and catalyze decarbonization pathways in the jurisdictions where the experimentation takes place *and* more broadly because of the interdependence of jurisdictions and systems locked into the use of carbon-based energy.

Here, we develop a framework to explore the efficacy and possibilities of disrupting carbon lock-in through subnational experimental pathways^3^ (Bulkeley et al. 2014; Hoffmann 2011; Jordan et al. 2015). It provides a way to makes sense of the means through which subnational experiments can catalyze and contribute to broader transitions to decarbonization. It identifies causal mechanisms that operate specifically along *political* pathways. Such a framework is needed because decarbonization pathways will not be constructed only through identification of economically efficient policy mixes, nor are they solely about adopting particular technologies or practices of energy production (cf. SDSN 2014; Global Commission on the Economy and Climate 2014). Instead, decarbonization implicates changes in social, technical, economic, and political systems that underpin modern societies. Put simply, whatever else it may be, disrupting carbon lock-in is fundamentally a political activity because lock-in has significant political foundations: It rests on norms, institutions, capacities, and coalitions that support fossil energy dependent systems. Pathways to decarbonization are thus paved with political decisions, policies, and voluntary initiatives that promote, alter, enable, constrain, and sometimes demand technological and behavioral changes. The framework developed here offers a new conceptualization of transformation toward decarbonization and an empirical strategy to explore how subnational experiments can catalyze change by altering political dynamics within and across jurisdictions, markets, and/or carbon-intensive practices.

We begin with a brief discussion of the nature and landscape of subnational experimentation and its evolving relationship with the global political response. We then develop our analytical framework that illuminates how diverse and targeted subnational experimental interventions^4^ interact with and alter the politics of carbon lock-in in specific places^5^ (e.g., jurisdictions, networks like a particular city network, or markets) *and* how those interventions can contribute to broader disruption and transformation.

We posit that once initiated, subnational experiments can alter political dynamics through three mechanisms: catalyzing normative change (normalization); building capacities to act differently, whether by mobilizing resources directly or via institutional change; and coalition building. These mechanisms determine whether and how the policies and practices that experiments promote *scale up* and *entrench* in the jurisdictions and systems being targeted for disruption (direct effects), as well as how they influence other jurisdictions and systems beyond the direct scope of the intervention (interdependent effects). These political dynamics generate three possible trajectories for direct and interdependent targets for disruption: unintentional reinforcement of carbon lock-in; improvements or efficiency gains in carbonized systems; or transformational decarbonization, a phase change whereby fossil energy (and/or other GHG generating processes) is not just lessened, but a new trajectory toward replacement or zero use of carbon-based energy is generated. We illustrate the framework’s utility with three short vignettes analyzing experimental trajectories before concluding with thoughts on how our approach can illuminate the relationship between experimentation and the global response to climate change.

## Subnational experimentation and the shifting global response

There is no consensus definition of climate governance experiments or experimentation. Investigations of experimental governance are diverse, but they all depart from similar observations: (1) the traditional approach to climate governance, multilateral treaty-making, is no longer sufficient (if it ever was) for addressing the problem; and/or (2) multiple actors are now engaged in climate governance that is only loosely connected to UN-based processes. From this baseline, authors’ characterizations of *experimentation* in climate governance run the gamut from controlled laboratory-like experimentation to more metaphorical ideas of trial and error with new approaches.^6^

All understandings of experimentation share the notion that something new is being tried out—there is a conscious intervention that differs from the status quo. Novel action is a constitutive element of experimentation. Beyond this commonality, however, approaches diverge. For some, experimentation is a worldview or philosophy and the novelty is the form of governance itself. Experimentalist governance is an observed or proscribed commitment to a process of trying something new, evaluating the results, and revising based on what was learned (e.g., Overdevest and Zeitlin 2014; De Búrca et al. 2014). Others focus on novel specific practices, like technological innovations or policy experiments or urban living labs (e.g., Voytenko et al. 2016). Still others focus on novel sources of rulemaking and authoritative actors (e.g., Hoffmann 2011; Castán Broto and Bulkeley 2013; Bulkeley and Castán Broto 2013).

Approaches can also be distinguished by how they characterize the process or practice of experimentation. Abbott (2017) characterizes experiments as formal or informal depending on the level of conscious experimentation and control over the process. Scholarship tends to view this as a continuum. At the “formal” end are those who reserve the label climate governance “experiments” for analogues of controlled laboratory experiments (Abbott 2017). These approaches tend to be proscriptive (there are no pure empirical examples of this kind of activity), suggesting that climate governance experimentation is a way for jurisdictions to be innovative and adaptive to climate change and focus on learning from policy initiatives and evaluation (e.g., Huitema et al. 2011).^7^ At the other end is a more metaphorical understanding that views climate governance experiments as novel attempts at governing climate by non-traditional global actors (Hoffmann 2011). Other approaches lie somewhere between (De Búrca et al. 2014; Castan-Broto and Bulkeley 2013) depending on how self-conscious the experimentation is and how much of a focus there is on learning from policy innovations and initiatives.

Climate governance experimentation, however conceived, has exploded in the last 10–15 years (e.g., Bulkeley et al. 2014; Andonova et al. 2017; Hoffmann 2011). Subnational actors and initiatives have been at the forefront. Municipal climate action and transnational city networks were some of the first experimental climate governance initiatives recognized in the literature (Betsill and Bulkeley 2004). These networks have grown in number, size and visibility (Acuto and Rayner 2016). For instance the C40 Group of large cities now includes 83 affiliated cities that collectively contain 1/12 of the world’s population (www.c40.org). Individual city-based action is now standard in much of the world, though perhaps more in the Global North than Global South. In just one study, Castan-Broto and Bulkeley (2013) catalogued 627 municipal experiments in 100 cities.

More broadly, scholars and international organizations have identified subnational experiments as part of the “groundswell”^8^ of climate activity that arose in the post-Kyoto Protocol period (Chan et al. 2015). The number and diversity of subnational experiments is impressive.^9^ Subnational emissions trading systems have proliferated with trading in California, Quebec, Ontario, and Chinese municipalities coming online in the last few years. Subnational initiatives around renewable energy and carbon markets in North America are also prominent sources of this “new” climate action (Rabe 2007; Stokes 2013). Meanwhile, US States and Canadian provinces are even engaging in de facto foreign climate policy. Multiple states and provinces formed or joined subnational networks like the Western Climate Initiative and the New England Governors and Eastern Canada Premiers Climate Action Plan. California has been especially proactive, signing memoranda of understanding to cooperate on climate policy with multiple subnational governments and nation-states.^10^

The relationship of these subnational efforts to the more formal global response to climate change has evolved over time. Subnational action prior to 2005 tended to be in preparation for the implementation of (not to catalyze) the policy changes expected to accompany global treaty-making (Betsill and Hoffmann 2011; Hoffmann 2011). For instance, the inclusion of market mechanisms in the Kyoto protocol negotiations and text catalyzed early national, subnational, and private experiments with emissions trading and carbon accounting. Nongovernmental organizations (NGOs), corporations, states, and subnational governments experimented in order to build capacity to function within a global emission trading regime the Kyoto Protocol was supposed to create (Betsill and Hoffmann 2011).

As it became clear that traditional multilateral climate governance would fail to deliver an effective global response (Victor 2011; Depledge 2006), experimental initiatives emerged as a key alternative. Many of these initiatives, alternatively labeled experimental, transnational, or polycentric (e.g., Bulkeley et al. 2014; Jordan et al. 2015; Cole 2015), were explicitly engaged in making rules. A range of actors thus began to act as governors (Avant et al. 2010) in the climate change governance space independent of the multilateral treaty-making process or national regulatory measures. Governance took multiple forms including cities forming transnational networks (Betsill and Bulkeley 2004; Bulkeley and Kern 2006); US states and Canadian provinces cooperating on climate agreements (Selin and VanDeveer 2005; Rabe 2004, 2007, 2008); and NGOs and corporations forming alliances to implement climate friendly technology in cities and regions.

Experimental governance was a decentralized and bottom-up response to climate change where the global regime was centralized and top down. It engaged diverse actors in new arrangements working on a full range of climate-related activities rather than being a state-centric attempt to distribute greenhouse gas emission reductions. The key questions in both academic circles and beyond, then, were less about how subnational experiments could catalyze action in the global regime, and more about where they came from, how they functioned and were organized, and whether they could be a *viable* alternative to the multilateral regime (Hoffmann 2011; Bulkeley et al. 2014; Meckling et al. 2015; Jordan et al. 2015).

The current context where broad decarbonization is the goal, with multiple actors and processes now being legitimized and recognized as climate governors, has changed the conversation once again. This context raises a key new analytic challenge: to understand how experimental initiatives can catalyze (generate and accelerate) decarbonization pathways.

## The politics of decarbonization

There are two ways to think about decarbonization. One is to start with the end point or goal and then develop a set of policies and technological combinations that would hypothetically, if implemented, get you there. This is the strategy of deep decarbonization (SDSN 2014) and socio-technical transitions (e.g., Geels 2002; Geels et al. 2004) approaches. For them, politics is what helps or prevents the technologies or strategies from being adopted (Meadowcroft 2007, 2009, 2011; Shove 2010; Shove and Walker 2007; Jordan 2009; Geels 2014; Turnheim et al. 2015). The other way is to start with the experiments and then analyze the politics they produce that can lead to different pathways. We choose the latter, while recognizing that any new initiative must disrupt the existing politics of carbon lock-in. Thus, our starting point is the way in which specific, on the ground governance and policy experiments *generate* political dynamics and how those political dynamics might lead to decarbonization in specific places and more broadly—how politics shape decarbonization pathways and possibilities. The problem of decarbonization is a problem of politics within and between multilevel spaces and practices where the politics of decarbonization play out in the global system (i.e., political and economic activities that can occur at, or cut across, jurisdictions or geographies of cities, provinces, regions, and nation-states).

The political challenge of decarbonization is to disrupt the interdependent, overlapping, reinforcing dynamics that lead to the continuing use of fossil energy across scales (the structural features on which socio-technical transition scholars tend to focus). Cities are locked into the use of fossil fuels because (among other reasons) of how they are physically planned, the practices of citizens around transportation and energy use, powerful political coalitions and incumbent interests, institutional capacities that make cities run politically, and the range of technological options available to city dwellers. Likewise, nation-states are locked into the use of fossil fuels because of similar (not the same) cultural, economic, political, and technological dynamics on a larger scale (i.e., national energy and transportation policy, political coalitions and powerful interests, national culture). Further, the lock-in in cities reinforces state-wide lock-in and global lock-in just as global lock-in reinforces state-wide lock-in and in turn lock-in at the municipal level.

We focus on the *political* aspects of carbon lock-in because no matter where you look—markets, cities, subnational jurisdictions, or nation-states—there are institutional and normative processes, and structures (political factors) contributing to carbon lock-in. The substance and functioning of the political factors differs across levels variously defined—municipal politics and national politics are obviously not the same, nor is the politics of commodity production and consumption the same as the politics of finance or the airline industry—but they similarly serve to reinforce carbon lock-in in all parts of the system. This conceptualization of the challenge of the politics of decarbonization generates two important parameters for theory building and analysis.

First, it implies that changes in specific places as well as the carbon locked-in system as a whole can be analyzed with a common analytic framework. This does not imply that the politics in these different systems or places are the same, it means that a single framework focused on politics can be used to make sense of carbon lock-in and attempts to disrupt it anywhere. The politics that reinforce and seek to disrupt carbon lock-in in transnational city networks can be *analyzed* the same way as the politics that reinforce and seek to disrupt carbon lock-in in provinces (though the way those politics play out are substantively very different). In each case, the political dynamics of normalization, capacities, and coalitions are at play both in terms of reinforcing carbon lock-in and in attempts to disrupt it.

Second, the multiple levels of carbon lock-in are interdependent—the politics of carbon lock-in and its disruption in transnational city networks are connected to the politics of carbon lock-in and its disruption in provinces and nation-states. This implies the need to uncover mechanisms that mutually link or assimilate the local to the global—how actions and outcomes in specific places can catalyze broader transformation (or stymie it)—to account for change and to show how changes at different scales implicate changes more broadly (Geels 2010). Thus moves toward decarbonization in multiple specific subnational experiments can and should be analyzed for *both* their specific effects on targeted jurisdictions and practices and their potential to catalyze broader transformation elsewhere.

### The political pathways of decarbonization

**Fig. 1:**
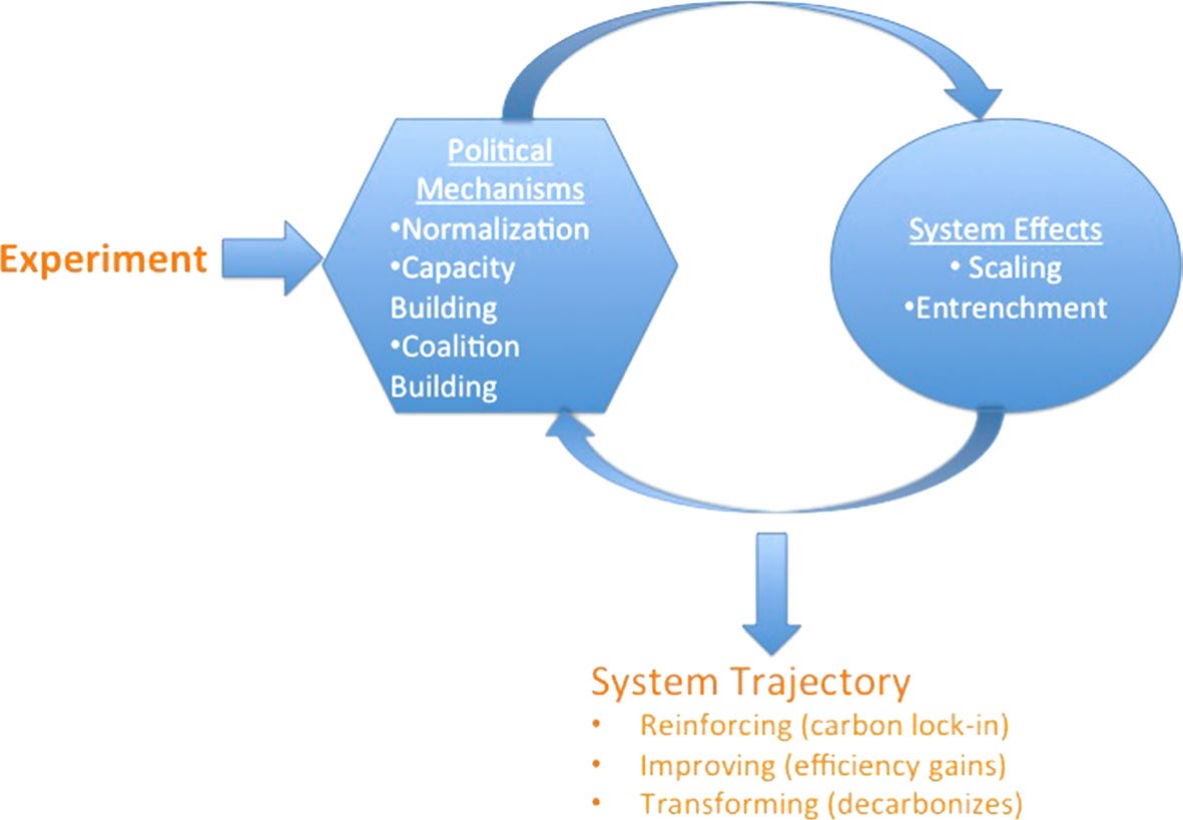
Decarbonization pathway in a targeted part of the system

Our analysis starts with an experiment, a conscious intervention designed to disrupt the current state of the targeted system.^11^ Once an experiment is initiated, the targeted system can move along one of the three (ideal-type) trajectories: (1) reinforcement of carbon lock-in, (2) improvement in carbon lock-in, or (3) decarbonization.^12^ The experimental intervention, whatever else it is, is political, and it contributes to changing the trajectory of the target by creating and/or contributing to political mechanisms of *normalization*, *capacity building*, and *coalition building*. These mechanisms help to determine whether the changes the experiment promotes will *scale up* and become *entrenched* in the targeted system, whether directly because the intervention itself grows, diffuses, and/or becomes institutionalized or because its policies and practices take on a life of their own, spawning further interventions or scaling and entrenching in other ways (changing other institutions, creating new legislation, altering business practices, etc.). Figure 1 provides a visual representation of this dynamic in a single place. Crucially, the potential for altering the target’s trajectory is found in the feedback between the experiment and the political mechanisms that it catalyzes.

**Fig. 2:**
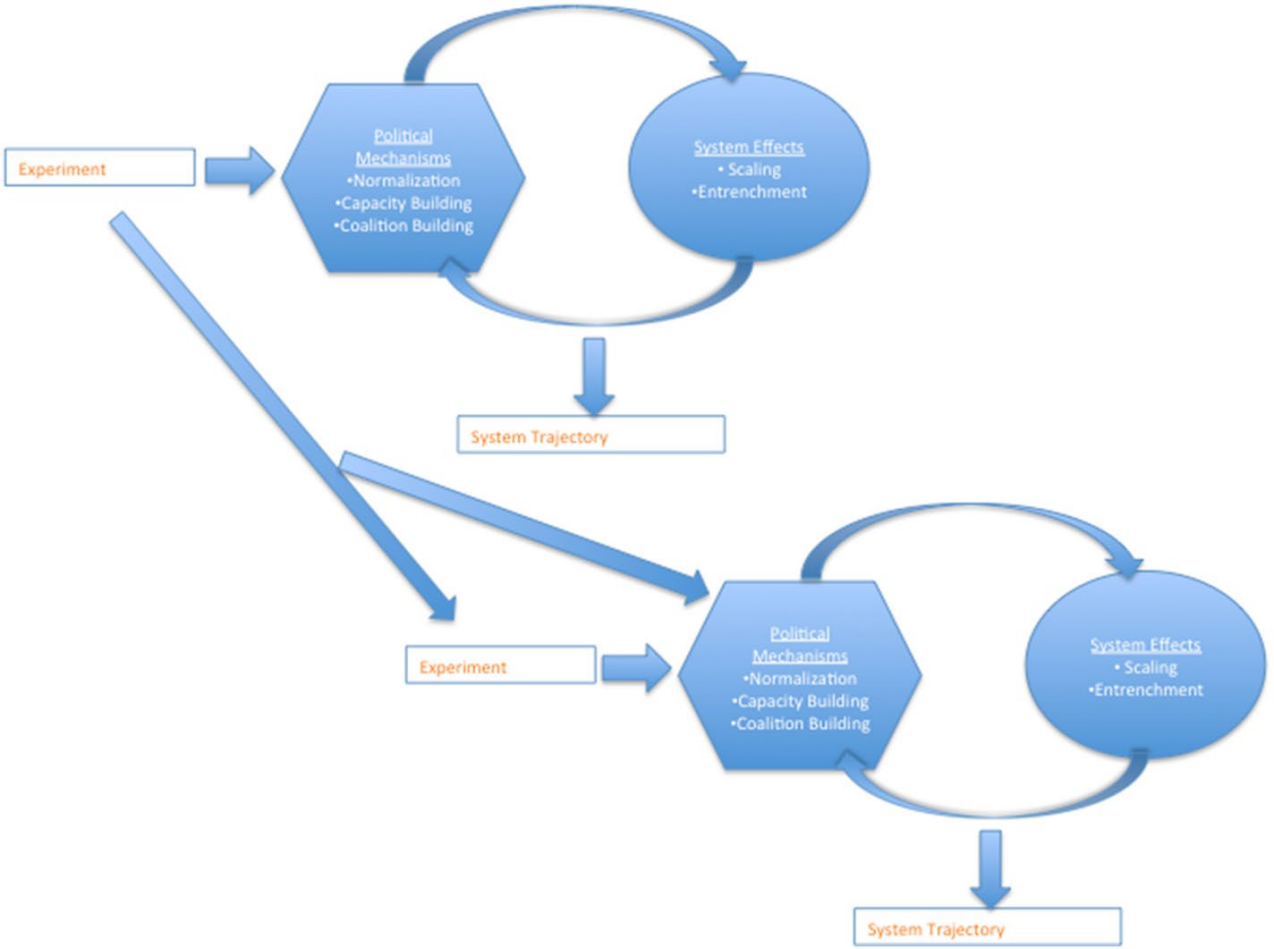
Decarbonization pathways across sub-systems

The interdependent nature of carbon lock-in, however, means an intervention in one place can also alter the politics in other places (Fig. 2). This crossover impact emerges in two ways. First, it is felt when an intervention in one part catalyzes the emergence of new interventions targeting other parts. For example, the C40 network of cities committed to combating climate change emerged, in part, in response to what was seen as a lacuna in the main existing transnational city network at the time (ICLEI’s Cities for Climate Protection). Second, an experiment in one system can contribute to the political mechanisms at play in other systems that were catalyzed by extant interventions. For example, subnational emissions trading systems in California and Quebec reinforced one another, eventually became linked and helped support the development of a system in Ontario that will join them. These broader impacts are of most interest to those considering orchestration—how and when can experiments catalyze the ‘right’ kind of other experiments and work together toward a common goal—and those thinking about how subnational experiments connect to NDCs (Hale and Roger 2014; Hale 2016; Abbott 2017). In the following subsections we elaborate on the different parts of the framework with illustrative examples drawn from research on multiple cases of subnational experiments.^13^

#### Unit of analysis: governance experiments

The fundamental unit of analysis when using this framework is the climate governance experiment—an initiative that seeks to disrupt carbon lock-in in a specific target through intentional attempts to authoritatively steer actors. Here, we focus primarily on subnational experiments but the logic of the framework applies to multiple kinds of initiatives. The catch, for rigorous case selection, is that there are no cases of wide scale (and only a few cases of small scale) decarbonization to compare with failed cases. Further, decarbonization is not a defined end state beyond the banal and obvious vanishing use of fossil fuels—we do not know what decarbonized systems will look like in any detail. Finally, the world is now awash in climate policies, emission reduction plans, low-carbon pilot projects, among other efforts combat or adapt to climate change, from multiple diverse subnational actors. This empirical context thus provides little in the way of definitive criteria to pick experiments to analyze.

One answer—the strategy we pursue in a larger project^14^—is to follow a diverse case selection strategy and include a large range of initiatives that vary in terms of initiating actor (public, private, hybrid), target (jurisdiction, market, practice), scope (from interventions that target specific activities like LED lighting in streetlights to interventions that focus on a combination of activities like renewable energy policy at the state/provincial level) and scale. Since our purpose here is mainly to introduce our framework and show its utility for analyzing subnational climate governance experimentation, we select subnational case vignettes and examples from our broader project to show how it can be applied as opposed to presenting a complete set of findings.

#### Targets

For simplicity’s sake, we identify three ideal types of targets on which experiments could focus in seeking to disrupt carbon lock-in and catalyze decarbonization trajectories:

1. *Political jurisdictions* Experiments may target individual polities (cities, states or provinces, countries), multiple jurisdictions horizontally (e.g., C40 or the 2014 China–U.S. agreement), or vertically (e.g., provinces and nation-states in a federally coordinated cap-and-trade system).
2. *Markets* Targeted markets can be sectoral (e.g., an experiment aimed at the airline industry) or jurisdictionally bounded (e.g., carbon labeling that targets supermarkets in a particular country). The distinguishing feature is that the intervention targets corporations, investors, consumers or other market actors and their practices directly.
3. *Practices* Practices are often less bounded than other targets. Relevant practices can be behavioral (e.g., cycling or energy conservation initiatives), cultural (climate fiction), and/or material (e.g., zero-carbon building design, consumer behavior, engineering training).

These targets are not mutually exclusive; there may be overlap and nesting. For example, a practice such as zero-carbon building design may be jurisdictionally bounded (e.g., a demonstration project in a particular city) or can cross boundaries via a transnational community of practice such as professional architects.

#### Political mechanisms

The three political mechanisms on which we focus are distilled from a broad reading of the politics of systemic change. Each one represents multiple literatures and theoretical approaches. Our attempt here is to provide a coherent framework that draws on a foundation of multiple strands of the political science literature as opposed to a generalizable causal model.

Norm change is often identified as an important source of shifts in public policies and interests, even if their effects are mediated by local politics and institutions (e.g., Keck and Sikkink 1998; Finnemore and Sikkink 1998; Meyer et al. 1997; March and Olsen 1998; Acharya 2004; Bernstein and Cashore 2012). Similarly, transition scholars have noted the potential for reframing at both niche and landscape levels to generate “higher-level changes in social norms and values” (Upham et al. 2014, 790). *Normalization* shifts expectations about appropriate behavior, thus, “If policy advocates succeed in generating a political and public expectation that [greenhouse gas] emissions should decline over time then policies and behaviors that further reduce GHG may be judged ‘better’ and more appropriate than those that engender increases” (Selin and VanDeveer 2005, 371–72).

Two mechanisms of norm change are particularly salient for our framework. First, entrepreneurs can propose and advocate new ways to look at the world and act on problems like climate change, catalyzing norm change (Finnemore and Sikkink 1998; see also Kingdon 1995 and Young 1991 on agenda setting). They reframe notions of appropriate action, work to convince others and alter the common sense of a system. Second, the buildup of everyday action on climate change—practices—can shift perceptions of the necessity and appropriateness of climate action; what people do “determines what they think” (Pouliot 2011, 21). The practices that experiments entail can shape how actors in different parts of the system, and ultimately society at large, understand climate change and their interests in taking aggressive action.

Decarbonization experiments can activate both of these mechanisms. Many interventions are entrepreneurial efforts that work on developing new practices of climate responses. For example, the Carbon Disclosure Project (now known simply as CDP) advocates for companies to account for and disclose their carbon emissions and exposure to climate risk. In response, many large corporations including GE, Google, Microsoft, and even Exxon have changed their practices and now engage in shadow pricing: they assume there will be a carbon price in the future and include the cost of carbon in their business planning (CDP 2013). The practice of treating carbon pricing as inevitable contributes to normalizing potential moves toward decarbonization in the corporate community and generates political support for public moves toward carbon pricing (Clark 2015).

Subnational experiments often seek to generate normalization very consciously in terms of setting standards to strive for. A municipal experiment in Toronto that has sought to develop a sustainable neighborhood on the waterfront actively attempts to normalize building practices that are greener than those driven by the building standards of Ontario.^15^ Real estate developers are the particular targets for these efforts. The main tool Waterfront Toronto is using to achieve normalization is the Minimum Green Building Requirements (MGBR). The MGBR are included as mandatory requirements in the development proposal requests issued by Waterfront Toronto, which means that developers know what they are bidding on and understand  that they will have to meet the standards.^16^ The MGBR are then incorporated into development agreements.^17^ Integration into development agreements is the mechanism of enforcement for the standards. However, the impact of the MGBR extends beyond the property where Waterfront Toronto has direct control:

[Waterfront Toronto] can’t force [developers of privately owned land] to do anything, but [developers] understand that the buildings going up across the street will be LEED Gold or in some cases LEED platinum. If you don’t have a LEED gold building, and the customer is figuring out which building to buy in, you have to be competitive. So I think that’s really pushing the market towards higher performing green buildings.^18^

The second political mechanism, *Capacity Building*, operates through altering material, institutional, and cognitive capacities to act on decarbonization (e.g., Weible and Sabatier 2014; Pierre and Peters 2000; Bernstein and Cashore 2012; Selin and VanDeveer 2005). Direct means through which interventions can increase capacity include, “direct funding, education, training, [technical] assistance, and… co-governance via partnerships between public and private actors and authorities” (Bernstein and Cashore 2012, 593). Similarly, capacity can be built via demonstration effects that act as policy learning vehicles (Selin and VanDeveer 2005; Rabe 2008). Interventions generate institutional capacity when they alter how governments make decisions and implement programs.

The electric vehicle pilot project of C40 nicely illustrates these mechanisms. C40 created a new institution, the Electric Vehicle Network, comprised of a subset of C40 cities as a first step. This network provides constituent city officials with detailed information regarding the benefits of pursuing an electric vehicle strategy and nudges them toward a particular approach to unlocking local demand through the creation of a robust and extensive charging infrastructure.^19^ In addition, the Electric Vehicle Network serves as a demonstration project, facilitated by C40’s city–city interaction and sharing of best practices (SLoCaT 2014).

Capacity building can move across parts of the system as well. For instance, the Carbon Registry (a California-based experiment that developed greenhouse gas accounting methods) has provided information and expertise for multiple actors looking to account for carbon, including transnational city networks, US states (Massachusetts and California), and nation-states (United States and Brazil).^20^ In this sense, one subnational experiment, as per figure two, influences the political mechanisms and landscape in other experiments and jurisdictions.

Finally, *Coalition Building* and dynamics are foundational in much of political science. Here we are especially interested in how coalitions build and change to support or resist new initiatives—how, in other words, interventions can spur the emergence and strengthening of economic and political coalitions that back decarbonization. They can catalyze these coalitions by identifying and linking “winners” in the move toward decarbonization and neutralizing losers. This entails empowering actors who have an interest in climate change, building constituencies either through creating or altering incentives or by active social movement building, and utilizing larger market forces.

For example, efforts to promote renewable energy portfolio standards and feed-in tariffs are designed to create winners (renewable energy companies, consumers) that can become a political force for sustained and/or broadened action (though these coalitions often face countercoalitions) (Rabe 2007; Stokes 2013; Aklin and Urpelainen 2013; Jacobsson and Lauber 2006). Even more overtly, carbon pricing initiatives commonly build in revenue distribution or compensation to build support or fend off countercoalitions, as Australia did by including subsidies to impacted sectors and flexibility mechanisms in its 2008 carbon pricing scheme (Gordon 2015, 131, 133). Similarly, research has documented how regulations, standard-setting and registries can foster “Baptist-bootlegger” coalitions of activists and businesses already following good practices who want to be recognized and rewarded in the marketplace, which can increase support for strong regulation in a sector or the spread of standards/regulations to other jurisdictions (Vogel 1995; DeSombre 2000, 79; Levin et al. 2012).

These coalitions can evolve over time during experimentation. For instance, The Climate Group’s SMART 2020 experiment, which sought to increase uptake of information and communication technology (ICT) in cities to drive down emissions saw three important phases of coalition building (Tozer 2016). After building a successful supporting coalition focused primarily on the ICT industry, the SMART 2020 program found that there was no market uptake for the industry’s supply. This led to shift in target for SMART 2020 from industry supply of ICT for carbon abatement to cities as a market for ICT solutions. SMART 2020 built a new supporting coalition that also included individuals from municipal governments around the world. Industry was still involved in this new supporting coalition, and SMART 2020 sought to play a matchmaking and barrier-smoothing role between the two groups. In the third phase, an opposing coalition criticized the ‘smart cities’ approach as it existed at the time (including but not limited to SMART 2020) for its democratic deficit. In some ways, this opposing coalition has been folded into the broader ‘smart cities’ coalition, in that new coalition members from the social enterprise and open data sectors have brought a new focus on citizen engagement to the ‘smart cities’ coalition (Tozer 2016).

Interventions can contribute to normalization, capacity building, and coalition building around the substance of what they are trying to do (carbon labels, renewable energy, smart grids, etc.) both in the experiments’ direct targets and beyond, but the interventions only provide the *potential* for these mechanisms to generate scaling and entrenchment. These mechanisms do not function in a vacuum and other countervailing conditions and factors play a role in determining whether that potential is realized. Moreover, separating out these mechanisms is an analytic convenience. In practice, they interact. Sometimes, they produce synergies, for example, scholarship on social movements has shown how NGOs, like experiments in our framework, can catalyze coalitions by framing an issue in ways that allow disparate actors to see common interests and benefits, and through what we call normalization generating commitment to a longer-term campaign (Tarrow 2005; Levi and Murphy 2006). Other times, they work at cross purposes, for example, if states learn particular climate practices from one set of interventions that disrupt the coalitions that other kinds of interventions generate. This framework cannot specify a priori all the ways that the political mechanisms can interact, but it does provide a basis for making sense of the details of particular experiments and tracing how the political mechanisms operate in specific contexts.

#### System effects I: Scaling

When interventions successfully contribute to normalization, capacity building, and/or coalition building, the policies and practices they support have the potential to scale up. Scaling can take multiple forms. Most basically, climate governance experiments can produce *simple scaling*—initiatives and/or the policies they promote start small and then grow. Growth can be in terms of size and/or range of activities; interventions attract more members and resources, expand their geographic scope, or begin to undertake different types of activities. For example, the C40 Cities Climate Leadership Group began as the C20, an ironic homage to the G20. Not only has the C40 Cities Climate Leadership Group grown larger, it has also grown stronger—learning and demonstration effects within the network have enabled C40 cities to take the lead on climate change in a number of ways (Gordon 2013).

Ecosystems of interventions can also emerge and expand because interventions open up political and economic space for further activity. Intervention begets intervention in important ways. This kind of clustering effect facilitates *self*-*organized scaling* and has the potential to engender increasing returns to interventions—a dynamic whereby adding interventions reduces the barriers to further innovations and encourages the expansion of complementary activity. Clustering produces new niches that additional interventions can fill and opens up opportunities for cooperation and competition that produces more interventions (Hoffmann 2011: 73–75). The voluntary carbon market is a quintessential example. Once carbon offsets producers emerged, this opened up room for additional interventions to make the market work—offset and carbon credit registries, carbon standard-setters, carbon accounting. The entire voluntary carbon market is an ecosystem of climate governance interventions; each of its functions is made relevant by the functioning of others (Hoffmann 2011: 129–134).

Finally, conscious borrowing of ideas or policies is *modular scaling*. This looks like some classic versions of diffusion (e.g., Graham et al. 2012; Busch and Jörgens 2005) or what DiMaggio and Powell (1983, 151–152) call “mimetic processes.” A key example of modular scaling is the proliferation or similar forms of transnational city networks over the last two decades that bring municipalities together to work on climate change at the local level (Acuto and Rayner 2016; Betsill and Bulkeley 2004).

#### System effects II: Entrenchment

Processes of entrenchment, like scaling, can take multiple forms. Here we draw primarily from the path-dependency literature. While others have noted the disruptive potential of policy innovation and experimentation to policies that lock in carbon (Jordan et al. 2003), our interest is the mirror image of that dynamic: processes that make new initiatives and/or the policies or practices they promote “sticky” or difficult to reverse by triggering or reinforcing coalition building or broadening, normalization and capacity building. There are four primary processes of entrenchment^21^ (Levin et al. 2012; see also Hacker 2002; Mahoney 2000; Page 2006; Pierson 2004; and Thelen 2003).

- *Lock in* when policies and practices have immediate durability or stickiness, such as when legislation is passed.
- *Self*-*reinforcing* when the costs to reverse a policy or change instigated by an initiative rise over time.
- *Positive feedback* when an initially untargeted population joins an initiative and thereby reinforces the choices of the initial target population to be part of the intervention and/or policy.
- *Increasing returns* when the benefits to targets of an intervention increase over time.

Entrenchment may occur directly or indirectly. That is, it may result from direct targeting by the experiment and effects on the targeted population, or it may occur indirectly when the impacts of the experiment go beyond its original objective but still lead to durable changes that lead to decarbonization in another jurisdiction or because of knock-on effects in a related sector. This notion is comparable to the idea of modular scaling, but here the focus is on the durability and irreversibility of policies.

When focusing on entrenchment processes, it is equally important to pay attention to counterdynamics, including negative feedback, when, for example, targets of an intervention experience costs and organize against it (Jordan and Matt 2014, 230; Weaver 2010; Aklin and Urpelainen 2013). Attention to both positive and negative dynamics, especially the formation of countercoalitions, is important when analyzing indirect or unintended consequences in a forward-looking mode of analysis such as implied by this framework. It also provides an opportunity for analysis: attention to these processes directs our gaze to opportunities that arise in seemingly unrelated policies or experiments that can indirectly create positive entrenchment dynamics for decarbonization.

## Illustrative examples along three trajectories

To illustrate how this framework can work in practice, we present three schematic vignettes of experiments that illustrate the use of the framework. We chose these experiments both because they show how the political mechanisms and system effects identified in our framework combine and produce feedbacks, interacting with the substance of interventions to shape the trajectories of targeted systems, and because each illustrates a different trajectory: system reinforcing, system improving, or decarbonizing. The framework can be used to structure pathway narratives that tell the stories of the experiments. The goal of pathway narratives is to make sense of what the intervention ‘does’ and characterize its impact on the targeted system. The three political mechanisms (normalization, capacity building, coalition dynamics) combine with the two system effects (scaling and entrenchment) in a single framework that allows for analyzing how interventions can disrupt carbon lock-in in the targeted system and if/how the intervention has catalytic potential in other, linked places.

Scaling and entrenchment are observable implications of the political mechanisms at work and developing narratives also makes it possible to observe how scaled and entrenched policies and practices can feedback (positively and negatively) on the political mechanisms. Sometimes what is observable is change in the experiment itself and sometimes it is the effect on policies, even if the experiment itself remains unchanged or disappears. Changes in trajectories are observable implications of the disruption that does or does not occur as result of feedback between political mechanisms and system effects. Scaling and entrenchment in this sense are necessary, but not sufficient, conditions for developing transformative trajectories. It could be that an experiment that entrenches and scales fails to achieve its intended purpose of disrupting carbon lock-in and instead unintentionally reinforces it or only improves on carbon lock-in.

For each experiment, the framework offers the parameters for analyzing and monitoring the trajectory of the experiment, how it contributes to normalization, capacity building, and coalition building, and how those mechanisms do or do not produce scaling and entrenchment. Since the politics of decarbonization are contested politics, much of the analysis concerns the obstacles to transformation—entrenched (or incumbent in the language of the socio-technical transitions scholarship) interests and coalitions, the capacity to perform practices associated with carbon lock-in, and the common sense around carbon lock-in, and how the intervention alters or fails to alter those dynamics.

A range of methodological tools can be employed in the development of pathway narratives and the various processes of scaling and entrenchment outlined above are observable through careful qualitative analysis of individual interventions. This process begins with descriptive analysis, identifying the goals, content, and activities of the intervention to provide an initial sense of whether its substance augurs toward decarbonization. Next is the analysis of how the activities of the experiment contribute to the political mechanisms and scaling/entrenchment dynamics (see Table 1 for indicators of scaling and entrenchment) and how they disrupt extant dynamics in the targeted system. This work is a matter of process tracing using data gleaned from intervention documents, media reports, and where appropriate and possible, interviews with intervention participants and actors that interact with the intervention activities.

**Table 1:** Indicators of scaling and entrenchment in subnational climate governance. *Source*: Adapted from van der Ven et al. (2017)

Type of scaling	Indicator *Has the intervention*	Types of entrenchment	Indicator *Did the intervention*
Simple	Attracted more members, expanded in geographic scope, or accumulated more resources?	Lock-in	Use mechanisms that gave it immediate durability?
Self-organized	Inspired symbiotic interventions?	Self-reinforcing	Become more difficult to reverse over time?
		Positive-feedback	Attract non-target members thereby reinforcing the decisions of early adopters?
Modular	Been consciously emulated in a different context?	Increasing returns	Do the benefits to participants from the intervention increase when more participants are brought on board or the longer the intervention is in place?

The process tracing in the pathway narratives allows identification of key leverage points and the primary dynamics of scaling and entrenchment (internally and externally) that are operative in the specific context of the intervention in question. This provides a way to understand the linkage between the intervention’s activities and the trajectory of the target system(s) and to draw some conclusions about where that trajectory might head and why.

### Reinforcing carbon lock-in in Colorado’s new energy economy?

The experiment in this case was a suite of state-level policies to promote renewable energy in Colorado (Betsill and Stevis 2016). It began in 2007 with a focus entirely on traditional renewable energy (wind/solar), but by 2009 included natural gas as “renewable.” The initial substance thus had transformative potential, but over time the intervention contributed to, at best, a system improving, if not a lock-in reinforcing trajectory.

The key political mechanism/systems effect feedback in this case was the way in which coalition building dynamics over time led to entrenchment of the intervention in an unexpected way. When the New Energy Economy policy program emerged, the coalition that backed it included the usual environmentalist suspects along with some rural landowner and union interests (Betsill and Stevis 2016; Betsill 2016). However, over time Colorado Governor Bill Ritter saw the need to expand the coalition to both entrench the New Energy Economy program and continue to expand the renewable energy standard (from 10 to 30%) in the state (Betsill 2016). This need for an expanded supportive coalition led to an evolution in the substance of the intervention as backers sought to split the fossil fuel interests by redefining natural gas as a renewable energy.

This coalition building move was successful in that the New Energy Economy has survived an election and continues to be a driving force in Colorado energy politics (Betsill 2016). In addition, the move from coal to natural gas in electricity production has the potential to lower Colorado’s GHG emissions. However, the question is the trajectory of this intervention. The coalition building-entrenchment feedback dynamic has led to fossil fuel lock-in improvement at best (natural gas replacing coal) but may have even reinforced carbon lock-in by entrenching natural gas production as a key part of Colorado’s economy. The positive interpretation is that “a coalition involving natural gas could be seen as a short-cut down the decarbonization path, making it easier to take more aggressive action in the long-term” (Betsill 2016: 20).

### Improving carbon lock-in through the unintended consequences of carbon labeling^22^

In 2008, the UK government-sponsored Carbon Trust developed a carbon labeling scheme as a marketplace intervention.^23^ It designed the labeling effort to provide consumers with product level carbon footprint data (the amount of GHGs that go into the production and transportation of the products) in order to drive their choices toward low carbon purchases, catalyzing moves toward decarbonization.

The interaction of capacity building, indirect entrenchment effects, and modular scaling crucially shaped the trajectory of this intervention. While directed at consumers, this intervention built significant capacity for corporations to measure the carbon footprint of their products. The Carbon Trust’s methodology for product footprinting as a form of capacity building allowed the intervention to generate simple scaling in terms of the number of corporations and products footprinted and modular scaling as a number of countries beyond the UK (e.g., France, Japan, Korea and Thailand) took up carbon labeling, borrowing elements from its methodology and standard, Publicly Available Specification (PAS) 2050 (Shi 2010; Vergez 2011, 11).

However, the system improving trajectory came from unintended consequences. The theory of change for the Carbon Trust was consumer driven—labels shape consumer behavior—but largely failed to take off (Carbon Trust 2015). However, tangible changes resulted from corporate learning via capacity building—the methodologies developed helped corporations locate and measure GHG emissions in their supply chains and manufacturing processes. Capacity building thus led to indirect entrenchment—the labeling intervention activities have been entrenched in ways unanticipated by the designers—and the combination has generated a system improving trajectory (through improved supply chain and process management), reducing emissions but not necessarily moving beyond fossil fuel use.

### Decarbonizing copenhagen through multilevel action?

In 2009, the city of Copenhagen adopted a goal of carbon neutrality by 2025 with an interim goal of cutting emissions 20% below 2005 levels by 2015 (Copenhagen Municipality 2009; see also Copenhagen Municipality 2012, 2013). Its initial plan to achieve that goal included a suite of policy initiatives in multiple sectors: energy, transport, buildings, urban planning, adaptation, and public outreach. Copenhagen’s plan goes beyond the corporate emissions of the municipality and seeks to functionally decarbonize the whole city. In this experiment, the feedback between normalization and both entrenchment and scaling is potentially generating a transformative trajectory.

Fostering the normalization of decarbonization is an explicit goal in Copenhagen’s plan, both among the populace and business community actively promoting this normative position through a deceptively simple formula that targets economic actors and the general public:

- The Good Life = Sustainable Life and a Green City = Economic Growth.^24^

For economic actors, the strategy stresses the green economy. As a representative of a major Copenhagen business association observed, “everyone knows that they [companies from Copenhagen] are green.”^25^ To socialize the public and make the plan more participatory, in 2013 the city launched multiple initiatives aimed at citizen engagement and behavioral change (Copenhagen Municipality 2013).^26^ Here the narrative goes beyond green growth and green economy to livability and the idea of sustainability as the key to the good life.^27^

Normalization efforts have contributed to the entrenchment of decarbonization goals in both municipal policy and in economic planning (green growth). They have mainstreamed climate change policy to the degree that climate planning is becoming conflated with economic and urban planning, reinforcing the common sense around decarbonization. The decarbonizing trajectory that results, however, is dependent upon and generates a multilevel scaling strategy. Copenhagen works to sell its approach (and businesses) abroad to keep the momentum for decarbonization going at home. For example, New York City and Copenhagen signed an agreement in fall of 2014 to set up a Danish clean tech hub in New York designed to “help New York City capitalize on Danish tech savvy…while providing Danish companies access to the New York marketplace” (Watts 2014). In this sense scaling out—advocating for Copenhagen’s solutions in other jurisdictions—through both transnational networks (C40) and in bilateral relations (New York City-Copenhagen connections), enhances the normalization of decarbonization in Copenhagen. It is the feedback between scaling, entrenchment, and normalization that is pushing Copenhagen toward a potentially decarbonizing trajectory.

### Forward theorizing

As is clear from these vignettes, the framework itself is not a recipe for getting to transformation from an experiment. It is a coherent set of concepts and parameters that allows us to conceptualize, explain, and track system trajectories toward decarbonization (or, alternatively, reinforced or improved carbon lock-in). The forward theorizing strategy that is necessitated by this framework is similar to Levin et al.’s (2012, 130): “to identify possible policy interventions and reason forward to how the problem and interventions might unfold over time.” We are interested “in other possible and likely futures, and in determining the ways in which [an intervention’s] actions and the actions of others contribute—sometimes via unintended effects and consequences—to making some of them real’’ (Patomäki 2006, 12). This approach recognizes a commonplace observation in analyses of complex systems: feedbacks can be both positive and negative (Weaver 2010; Jordan and Matt 2014) and the effects of relationships of components of a system and political mechanisms can be indeterminate.

## Conclusion

We have this thing called the “cheerful disclaimer”—which means we have no idea if the idea is going to work or not. It’s an invitation to have a go. Rob Hopkins, Founder of Transition Towns.^28^

There are thousands of subnational experiments that are having a go. We need to update our conceptual apparatus for understanding what they are accomplishing and where they might be headed. Only then can we actively assess if they are going to work or not to turn aspirational goals into a decarbonized reality. Yet, we lack a consensual or proven means to grasp the impact of subnational activity. Our framework takes experiments seriously as a potential means to catalyze decarbonization trajectories, recognizing that their potential or trajectory generally cannot always be calculated a priori. Instead, it provides a way to identify and track the political forces and mechanisms through which experiments have an impact upon targets of intervention and make (or fail to make) broader connections.

This conceptual innovation can speak to questions, both academic and policy, about the relationship between subnational experiments and the UNFCCC and the NDCs that constitute the substance of the Paris Agreement. Understanding how subnational climate governance experiments catalyze decarbonization trajectories is a first step to grasp the possibilities and potential for orchestration of subnational initiatives through the UNFCCC and the role that such initiatives might play in ratcheting up NDCs.

Orchestrating experimentation (Hale and Roger 2014; Hale 2016; Chan et al. 2015; Abbott 2017) is often discussed as finding ways for the global regime to harness the characteristics of subnational experiments to further the goals of the UNFCCC and Paris Agreement, including monitoring and adaptation. “Orchestration platforms” (van der Ven et al. 2017) have emerged or are being designed to catalogue and spur subnational action—the Lima-Paris Action Agenda, NAZCA, the Groundswell, etc.—but it is not yet clear that these databases can capture the ways in which subnational action can have a global effect, to say nothing of how they can catalyze more effective global action. Key challenges in designing and implementing orchestration include figuring out how to evaluate the impact of experiments and how to generate synergies among them. Our framework can provide valuable guidance along these lines because it offers a way to understand impact beyond acontextual emissions figures and focuses directly on how interactions across experiments are already developing.

Our framework is also useful to better understand the relationship between subnational experiments and NDCs themselves. Experiments could have a direct or indirect effect on the stringency and ratcheting potential of NDCs. Canada, for instance, explicitly references provincial policy experiments with carbon pricing in its *Pan*-*Canadian Framework for Clean Energy and Climate Change* (2017: 6–7) that lays out its NDC implementation plan. In the United States, both before and after Trump’s announcement of the US intention to withdraw from the Paris Agreement, there was much talk and analysis of whether subnational efforts could keep the United States on track to achieve its Paris commitment. Our framework has advantages here precisely because it allows for analysis of direct impact—how subnational experiments can influence the development of national policies through the political mechanisms and system effects—and indirect impact—how experiments alter the broader political landscape and appetite for aggressive climate action.

## Footnotes

1 See, e.g., Climate Tracker’s analysis at http://climateactiontracker.org/global/173/CAT-Emissions-Gaps.html; United Nations Environment Program (2017).

2 In discussing subnational climate governance experiments, we are referring to both experiments initiated by subnational governments and experiments initiated by non-state actors that operate at the subnational level.

3 The framework is discussed in terms of subnational experimentation, the empirical focus of this article, but it can be applied more generally to explore the politics and impact of experiments and initiatives developed by diverse actors, including states, international organizations, “clubs” like the G20, partnerships, and the huge array of non-state actors engaged in climate governance activities.

4 Throughout this paper, we use the term “experiment” interchangeably with the term “intervention.”

5 We self-consciously use the generic “place” when referring to the location of experiments as opposed to “level” or “regime” since the latter terms have specific—and different—meanings in the political science, international relations, and transitions literatures.

6 For comprehensive reviews of the deployment of experiment, experimentation, and experimental as concepts applied to climate governance, see Kivimaa et al. (2017), and Bulkeley and Castán Broto (2013).

7 Much of this stems from studies of European policy experimentation see, e.g., Sabel and Zeitlin (2008).

8 http://www.climategroundswell.org/blog-test/lpaa/report.

9 http://newsroom.unfccc.int/lpaa/cities-subnationals/.

10 http://www.climatechange.ca.gov/climate_action_team/partnerships.html.

11 It is certainly possible to examine interventions that are not conscious attempts at decarbonization as a range of policy experiments might have decarbonization implications. This is beyond the scope of this article. In addition, we assume a broad definition of experimentation, including both the formal and informal ends of the spectrum discussed above.

12 We cannot know at the outset whether or not the implications and results from the experiment are transformative. Our framework is meant to be open to these possibilities—that even experiments meant to disrupt carbon lock-in can end up reinforcing it. Experiments can and do fail.

13 These cases are drawn from a larger project on the politics of decarbonization (https://munkschool.utoronto.ca/egl/researches/transformative-policy-pathways-to-decarbonization/).

14 https://munkschool.utoronto.ca/egl/researches/transformative-policy-pathways-to-decarbonization/.

15 For a fully developed discussion of this experiment, see Tozer (2018).

16 Interview with Waterfront Toronto employee, August 19, 2015.

17 Interview with Waterfront Toronto employee, August 19, 2015.

18 Interview with Waterfront Toronto employee, August 19, 2015.

19 Interview with Steve Crolius, former Director of Transportation, Clinton Climate Initiative.

20 http://www.theclimateregistry.org/thoughtleadership/greenhouse-gas-accounting-verification/. Accessed May 14, 2015.

21 These processes are not mutually exclusive. All can be at play and they can overlap. They are separated out here for analytic convenience.

22 This is not technically a subnational experiment in the same way as municipal action, but this case shows the generality of the framework and how political dynamics within the state can go transnational.

23 For a fully developed discussion of this case see van der Ven et al. (2017).

24 Authors’ formulation based on readings of city documents and interviews with city staff and stakeholders.

25 Confidential interview, Copenhagen, April, 2014.

26 Confidential interview with municipal official Copenhagen, April, 2014.

27 Confidential interview with municipal official, Copenhagen April, 2014.

28 Rob Hopkins, founder Transition Towns experiment, quote taken from http://www.newscientist.com/article/mg20527466.000-rob-hopkins-getting-over-oil-one-town-at-a-time.html?DCMP=OTC-rss&nsref=online-news. Accessed February 9, 2010.
